# Generation of insulin-secreting cells from mouse gallbladder stem cells by small molecules in vitro

**DOI:** 10.1186/s13287-019-1407-6

**Published:** 2019-09-23

**Authors:** Fei Chen, Tuo Li, Yu Sun, Qinggui Liu, Tao Yang, Jiajia Chen, Haiying Zhu, Yongquan Shi, Yi-Ping Hu, Min-Jun Wang

**Affiliations:** 1 Department of Cell Biology, Center for Stem Cell and Medicine, Navy Medical University (Second Military Medical University), 800 Xiangyin Road, Shanghai, 200433 China; 2 Department of Endocrinology, Changzheng Hospital, Navy Medical University (Second Military Medical University), 415 Fengyang Road, Shanghai, 200003 China

**Keywords:** Gallbladder stem cells, Diabetes, Pancreatic β-like cells, Stem cell therapy, Differentiation

## Abstract

**Background:**

Stem cell-derived pancreatic β-like cells hold great promise for treating diabetes. Gallbladder belongs to the extrahepatic bile duct system and possesses stem-like cells. These stem cells could be expanded in vitro and have the potential of differentiating into hepatocytes, cholangiocytes, or pancreatic cells. As the gallbladder is highly available, gallbladder stem cells provide a new cell source of pancreatic β-like cells. In this study, we aimed to investigate an approach for the generation of pancreatic β-like cells from gallbladder stem cells (GSCs) without genetic modification.

**Methods:**

A CK19Cre^ERT^;Rosa26R-GFP mouse was used to isolate CK19^+^ cells, which represented EpCAM^+^ stem cells in the gallbladder. They were cultured in the modified Kubota’s medium for expansion and further analyzed. Then, we developed a strategy to screen a combination of small molecules that can generate insulin-secreting cells from gallbladder stem cells. These cells were identified with markers of pancreatic cells. Finally, they were seeded into the cellulosic sponge and transplanted to the diabetic mice for functional examination in vivo.

**Results:**

Gallbladder stem cells could be expanded for more than 15 passages. They expressed typical hepatic stem cell markers including CK19, EpCAM, Sox9, and albumin. By screening method, we found that adding Noggin, FR180204, and cyclopamine could efficiently induce gallbladder stem cells differentiating into insulin-secreting cells. These cells expressed Pdx1, Nkx6.1, and insulin but were negative for Gcg. After transplantation with the cellulosic sponge, they could ameliorate hyperglycemia in the diabetic mice.

**Conclusion:**

This study provides a new approach which can generate insulin-secreting cells from the gallbladder without genetic modification. This offers an option for β cell therapy in treating type 1 diabetes.

## Background

The gallbladder belongs to the extrahepatic bile duct system, which comprises the common hepatic duct, the bile duct, and the common hepato-pancreatic duct [1]. The gallbladder epithelial cells express epithelial cell adhesion molecule (EpCAM), CD49f, cytokeratin 19 (CK 19), Hnf1b, Sox9, and Sox17 [2–6]. These cells were proved to exhibit characteristics of multipotent stem/progenitor cells. They could be expanded in vitro for the long term and could differentiate into functional cholangiocytes, hepatocyte-like cells, and even pancreatic β-like cells.

Both the gallbladder and the pancreas are part of the endoderm lineage and are derived from committed progenitors [1, 3]. Previous studies demonstrated that the gallbladder epithelial cell could be genetically reprogrammed to β cell fate in or ex vivo [3, 7]. Moreover, in the streptozotocin (STZ)-induced diabetic mice, the biliary tree stem cells were capable of differentiating toward pancreatic islet fates [5]. Therefore, the gallbladder epithelial cells were considered as a cell source for pancreatic β-like cells.

The aims of this study were to isolate stem cells from the adult mouse gallbladder and differentiate them into insulin-secreting cells without gene modification. Some signaling pathways which are important for pancreatic lineage specification or can promote pluripotent stem cells differentiating into functional pancreatic β cells have been documented [7–13]. Therefore, we could screen a combination of the chemicals to induce gallbladder stem cells toward the β cell fate. Previously, study showed that an epithelial surface marker, EpCAM, could be used to isolate the stem cell population in the gallbladder [4, 6]. The gallbladder stem cells can be cultured in Kubota’s medium (KM) [2, 4].

In our study, a CK19Cre^ERT^;Rosa26R-GFP mouse was used to isolate CK19^+^ cells, which represented EpCAM^+^ epithelial cells in the gallbladder and were proved to possess stem cells [6, 14, 15]. The GFP^+^ cells could grow in the modified KM medium and exhibited hepatic stem cell characteristics. By our screening strategy, we established a method with a unique combination of small molecules to generate insulin-secreting cells from gallbladder stem cells. These cells expressed typical markers of pancreatic β cells and exhibited functional characteristics in vivo. Furthermore, we provide a new option for cell transplantation by using cell sponge as a scaffold. The gallbladder is a dispensable organ and can be easily accessed through a minimally invasive laparoscopic procedure. Our study demonstrated the feasibility of this convenient strategy to generate insulin-secreting cells, which could be a promising source for cellular therapy of diabetes.

## Materials and methods

### Animal models

Male, 8–10-week-old C57BL/6J mice (Shanghai Model Organisms Center, Shanghai, China) and CK19^CreERT^;Rosa26R-GFP mice (Tongji University, Shanghai, China) were housed in specific pathogen-free (SPF) rooms under controlled temperature, exposed to alternating 12-h light/dark cycles. Mice were provided with sterile water and standard rodent diet. The study protocol was approved by the Institutional Animal Committee of the Second Military Medical University (Shanghai, China).

To label the CK19-expressing epithelial cells, CK19^CreERT^;Rosa26R-GFP mice were intraperitoneally injected with 400 μl tamoxifen solution (dissolved in 10 mg/ml corn oil, Sigma-Aldrich, St. Louis, MO, USA) for three times, every other day. Seven days after the start of the tamoxifen injections, the mice were sacrificed and the gallbladders were excised.

To establish the type 1 diabetic mouse model, C57BL/6J mice were intraperitoneally injected with 50 mg/kg STZ (Sigma-Aldrich) daily for 5 days. Blood glucose levels were examined from the tail vein blood with a blood glucose meter (Roche, Indianapolis, IN, USA) after fasting overnight, two times per week. The mice developed hyperglycemia within 5 days of the final STZ injection. The fasting blood glucose maintained at a high level (> 350 mg/dl), 4 weeks after the final injection. Mice were considered to be diabetic if their blood glucose measurements remained high at levels > 350 mg/dl for 2 weeks. Transplantation surgery was conducted on an established diabetic mouse with anesthesia using 2,2,2-tribromoethanol (240 mg/kg body weight, Sigma-Aldrich).

### Gallbladder tissue processing and cell isolation

After sacrificing the animals, the gallbladders of C57BL/6J and CK19^CreERT^/Rosa26R-GFP mice were dissected from the liver, cut into pieces, and washed with ice-cold Hanks’ Balanced Salt Solution. For immunofluorescence staining, the tissues were embedded in optimal cutting temperature compound (OCT, Sakura, Japan) and maintained at − 80 °C. For digestion, the tissues were cut into small pieces and incubated with 0.25% trypsin/0.1% EDTA (Thermo Fisher, Waltham, MA, USA) for 30 min at 37 °C to obtain a cell suspension.

Cells obtained from the digested gallbladders of CK19^CreERT^/Rosa26R-GFP mice were filtered through a 40-μm strainer and re-suspended in a single cell solution. The cells were then sorted with fluorescence-activated cell sorter (FACS) by the detection of the green fluorescent protein (GFP) on the fluorescein isothiocyanate fluorescence channel using FACS ARIA II (BD Biosciences, San Jose, CA, USA). Sorted GFP-expressing cells were cultured in Kubota’s medium (KM) for primary culture. For long-term culture, 5% fetal bovine serum (Gibco, Gaithersburg, MD, USA) was added. Limiting dilution experiments were performed to obtain purified colonies from the GFP-expressing cells. Two of these colonies were selected for further studies.

### Quantitative real-time PCR and reverse transcription PCR

Total RNA from the GSCs was isolated using the TRIzol reagent (Invitrogen, Carlsbad, CA, USA) according to the manufacturer’s instructions. Briefly, 2 μg RNA was reverse transcribed into cDNA using the SuperScript II Reverse Transcriptase (Invitrogen) according to the manufacturer’s instructions. Mouse-specific primers were designed by Oligo 7 (Additional file 1: Table S1). Quantitative real-time PCR was performed in three repeats of each sample using the ABI-7900 system (Applied Biosystems, Foster City, CA, USA) with the SYBR Green Master Mix (Applied Biosystems). Fold change was calculated using 2^−ΔΔCT^. Expression of mouse Gapdh was taken as a control and normalized to 1.

### Karyotype analysis

The karyotypes of cells were examined as previously described. Briefly, colchicines were added to the culture medium at a final concentration of 0.2 μg/ml. Four to 6 h after incubation, cells were harvested and treated with hypotonic (0.075 M KCL) for 25 min. Then, cells were treated with 3:1 (v/v) acetic methanol fixative, and fixed cells were dropped from approximately 35 cm to chilled slides. The chromosome of the cells was stained with Giemsa (Sigma-Aldrich). The slides were observed under a microscope. The spread chromosomes were counted in more than 30 individual cells [16, 17].

### Immunofluorescence staining

To locate and identify the hepatic cell populations, the C57BL/6J and CK19^CreERT^;Rosa26R-GFP gallbladder tissue sections were used to detect CK19 and EpCAM expression by immunofluorescence staining. First, the frozen sections were fixed with 4% PFA, blocked with 2% bovine serum albumin, and stained with primary antibodies (Additional file 1: Table S2) overnight at 4 °C. After incubation, the samples were rinsed with PBS Tween-20 for 5 min, and incubated with the corresponding secondary antibodies (Additional file 1: Table S3) for 30 min at 37 °C. The cells were fixed in 4% PFA, rinsed with PBS Tween-20, and blocked with 2% normal goat serum. Next, the fixed cells were incubated with the primary antibodies, followed by washing and incubation with the secondary antibodies. The sections or cells were counterstained with 4′,6-diamidino-2-phenylindole (DAPI) (Thermo Fisher, Waltham, MA, USA). The tissue sections and cell cultures were examined using a Nikon Eclipse 50i Fluorescence Microscope (Nikon, Tokyo, Japan).

### Lentivirus production and infection

A reporter cell line was developed to monitor insulin expression in the GSCs undergoing differentiation in vitro. Briefly, lentiviruses were generated in 293T cells with pMD2.G and pSPAX2 vectors and the Ins2-Promoter or CMV Promoter (OBio, Shanghai, China), respectively. The lentiviral particles were harvested 48 h after co-transfection and filtered through a 0.45-mm cellulose acetate filter (Millipore, Milan, Italy). The expression of insulin would result in the expression of mCherry, which was detectable with fluorescence microscopy.

### Induction of GSCs to insulin-secreting cells in vitro

To develop a strategy to differentiate GSCs into insulin-secreting cells, the KM was diminished with nicotinamide as a basic medium (without FBS). A number of compounds were selected as candidates including Noggin (25 ng/ml, R&D, Minneapolis, MN, USA), nicotinamide (10 mM, Sigma-Aldrich), LDE225 (5 μM, Selleck, Houston, TX, USA), LY294002 (10 μM, Selleck), FR180204 (2.5 μM, Selleck), retinoic acid (5 μM, Selleck), and HGF (10 ng/ml, R&D) based on previous findings. A two-round screening of the compounds was performed. In the first round, the basic medium was supplemented with combinations of candidates listed in Additional file 1: Table S4, which were reduced by one component in each well. In the second round of screening, the medium was supplemented with components that were found to be essential for the differentiation of cells in the first round of selection, and the other compounds were added to different wells (also listed in Additional file 1: Table S4).

### ELISA assay

Insulin levels in the cell culture medium were measured with an enzyme-linked immunosorbent assay (ELISA) kit (CUSABIO, Houston, USA). The procedure was performed according to the manufacturer’s instructions.

### Transplantation of gPB cells

After inducing the differentiation of GSCs into gPB cells, the cells were digested and seeded into a cellulosic sponge (Bio-Byblos Biomedical Co., Taiwan, China) with 1 mm thickness, 9 mm diameter, and 200 μm microporous at a density of 5 × 10^5^ cells within 1 day of culture. After 2 days, the sponges were implanted at the epididymal pad site in the diabetic mice. Normal C57BL/6J mice, diabetic mice with transplanted gallbladder cells, and diabetic mice without transplantation were used as controls. For each group, at least six mice were performed for the experiments.

### In vivo glucose tolerance tests

Diabetic mice were fasted overnight for 16 h before they were given glucose by intraperitoneal injection at a dose of 2 g/kg body weight. Blood glucose levels were assayed at 0, 15, 30, 90, and 120 min after administration at 4 weeks post-transplantation.

### Statistical analysis

All experiments were performed in triplicate with the data expressed as the mean ± standard deviation. Differences between the groups were analyzed with the paired *t* test or one-way analysis of variance as appropriate. Statistical analyses were carried out with GraphPad Prism 5.0 (GraphPad Software, La Jolla, CA, USA). *p* values < 0.05 were considered statistically significant.

## Results

### The gallbladder epithelium possesses a stem cell population

**Fig. 1:**
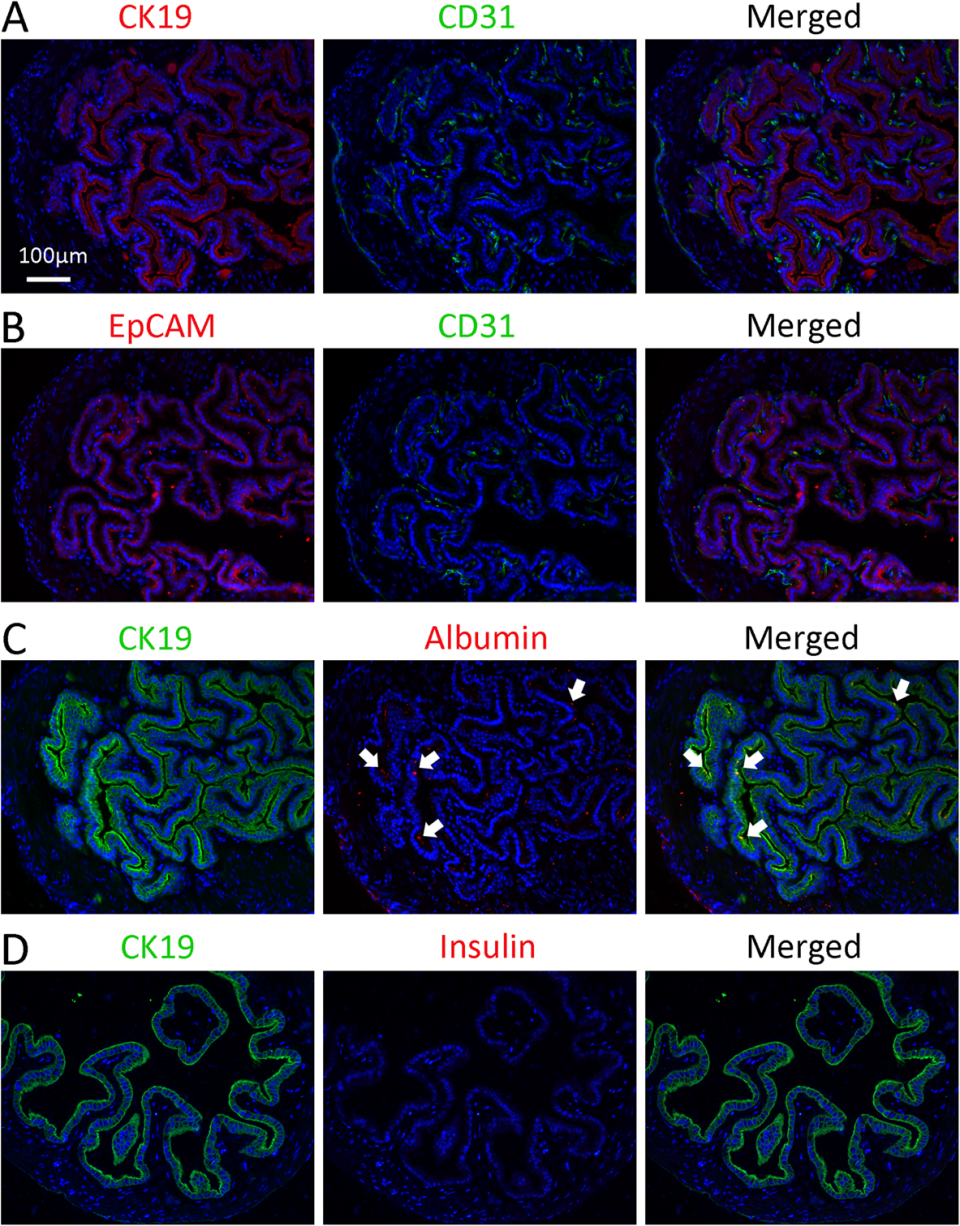
Immunophenotype of cells in normal mouse gallbladders. **a** Immunostaining of CK19 and CD31 in the gallbladder. **b** Immunostaining of EpCAM and CD31 in the gallbladder. **c** Immunostaining of CK19 and albumin in the gallbladder. Arrows indicate CK19^+^Albumin^+^ cells. **d** Immunostaining of CK19 and insulin in the gallbladder. No insulin^+^ cells were observed in the normal gallbladder epithelium cells. The nuclei were counterstained DAPI. Scale bar, 100 μm

Previously, study showed that the EpCAM^+^ cells could represent the population of gallbladder stem/progenitor cells [4, 6, 14, 15]. To locate and identify the stem/progenitor cell population, the gallbladder was collected from normal mouse and was examined by confirmed markers. CK19, a pan-biliary marker, was expressed by biliary epithelial cells along the bile duct system (included gallbladder) [14, 15, 18]. Immunofluorescence staining demonstrated that biliary epithelial cells were CK19^+^, EpCAM^+^ (Fig. 1a, b), and CD31^−^ (an endothelium cells marker). Interestingly, some epithelial cells also expressed albumin (a hepatic marker), and these double-positive cells were considered as stem cells previously (Fig. 1c, indicated by arrows) [16]. Furthermore, gallbladder epithelial cells did not express insulin (Fig. 1d). These results indicated that both CK19 and EpCAM could mark gallbladder epithelial cells.

### CK19 identifies a stem/progenitor cell population in the gallbladder

**Fig. 2:**
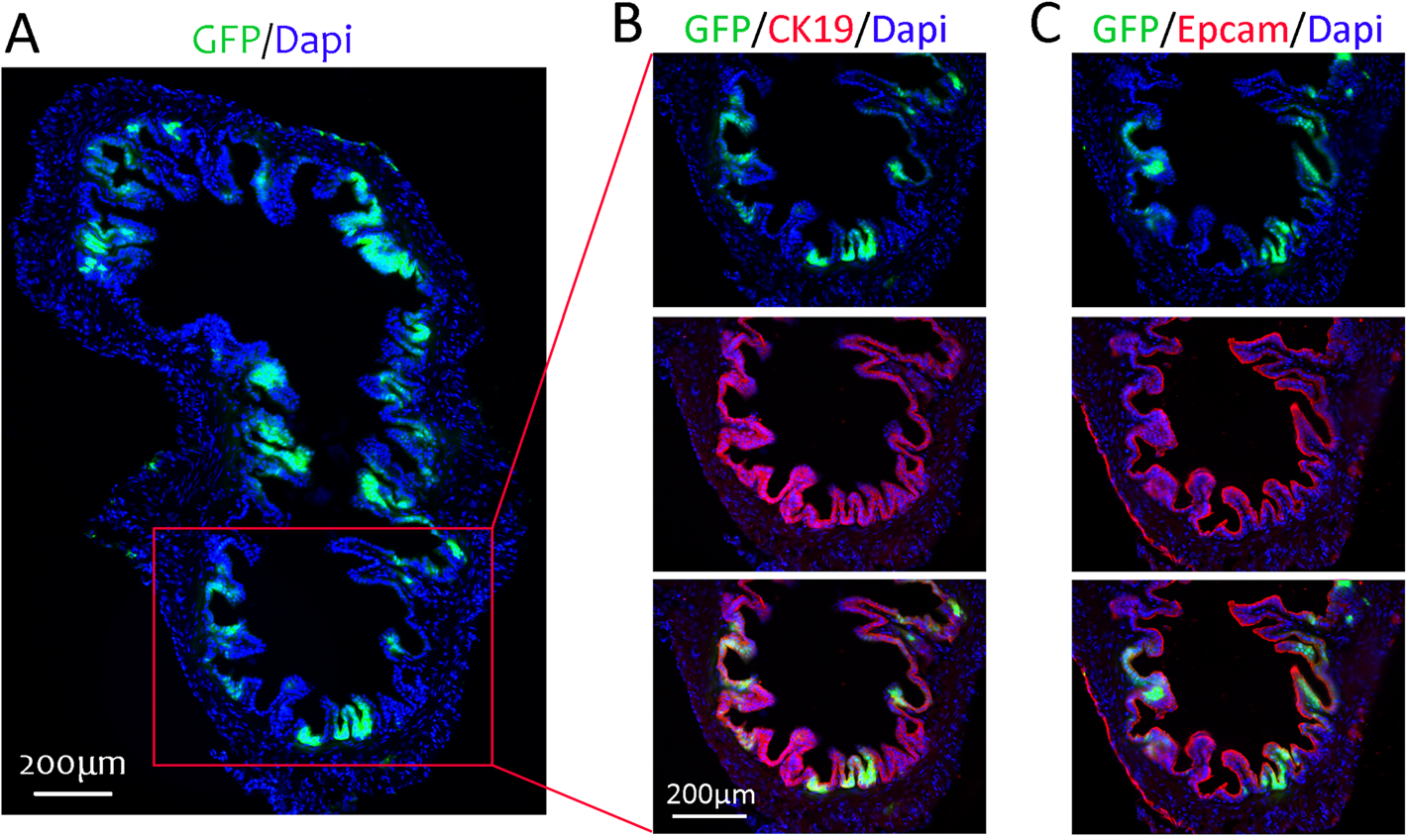
Immunostaining of CK19 and EpCAM in the gallbladder of CK19^CreERT^;Rosa26R-GFP mouse. **a** Total gallbladder in low magnification. GFP was observed by direct fluorescence. **b**, **c** Direct fluorescence of GFP with immunostaining of CK19/EpCAM. The nuclei were counterstained with DAPI. Scale bar, 200 μm

As CK19 marked the cell population overlapped with EpCAM, the CK19 was applied as the marker for isolating the potential stem cell population in our study [18, 19]. CK19Cre^ERT^;Rosa26R-GFP mouse could specifically mark the bile duct epithelial cells, and the CK19^+^ cells were labeled with GFP as previously reported [14, 15, 20]. Therefore, CK19Cre^ERT^;Rosa26R-GFP mouse was utilized in our study to isolate Krt19^+^ cells. Seven days after tamoxifen injection, the mouse gallbladder was collected and analyzed. The GFP^+^ cells were observed in the epithelium of the gallbladder (Fig. 2a). No GFP^+^ cells could be detected without tamoxifen (not shown). Co-staining revealed the GFP^+^ cells were also CK19^+^ and EpCAM^+^ (Fig. 2b, c).

**Fig. 3:**
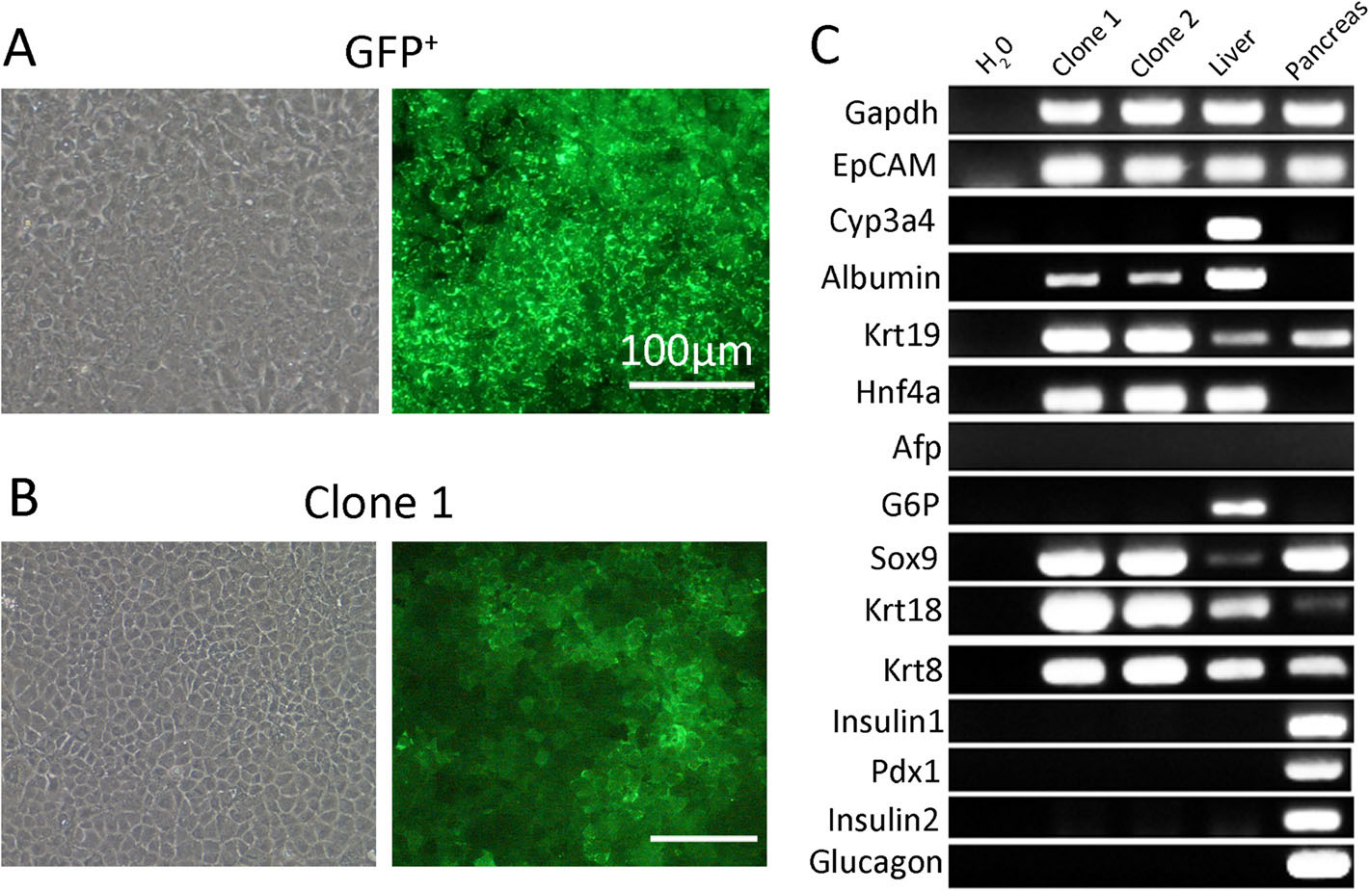
CK19^+^ cells exhibit basic characteristics of hepatic stem cells in vitro. **a** Phase contrast and fluorescence images of cultured GFP^+^ cells and representative single clone (**b**). Flat colonies consisted of epithelial cells. Scale bars, 100 μm. **c** RNA expression results for detection of liver-related genes and pancreas-related genes

Then the GFP^+^ cells were isolated by FACS and cultured in conditions that select for gallbladder epithelial cell growth. Results indicated that GFP^+^ cells could grow in high density, were smaller, and with a high nucleus to cytoplasmic ratio (Fig. 3a). Limiting dilution experiments were performed to obtain purified colonies from the GFP^+^ cells. Colonies were formed with small, polygonal, and tight cells (Fig. 3b). Two colonies were randomly selected for analysis and showed the cells were positive of stem cell markers, including CK19, EpCAM, Sox9, and albumin. They did not express a mature marker, such as Cyp3a4 and G6P. Also, the cells were negative for pancreatic lineage markers: Pdx1, insulin, etc. (Fig. 3c).

**Fig. 4:**
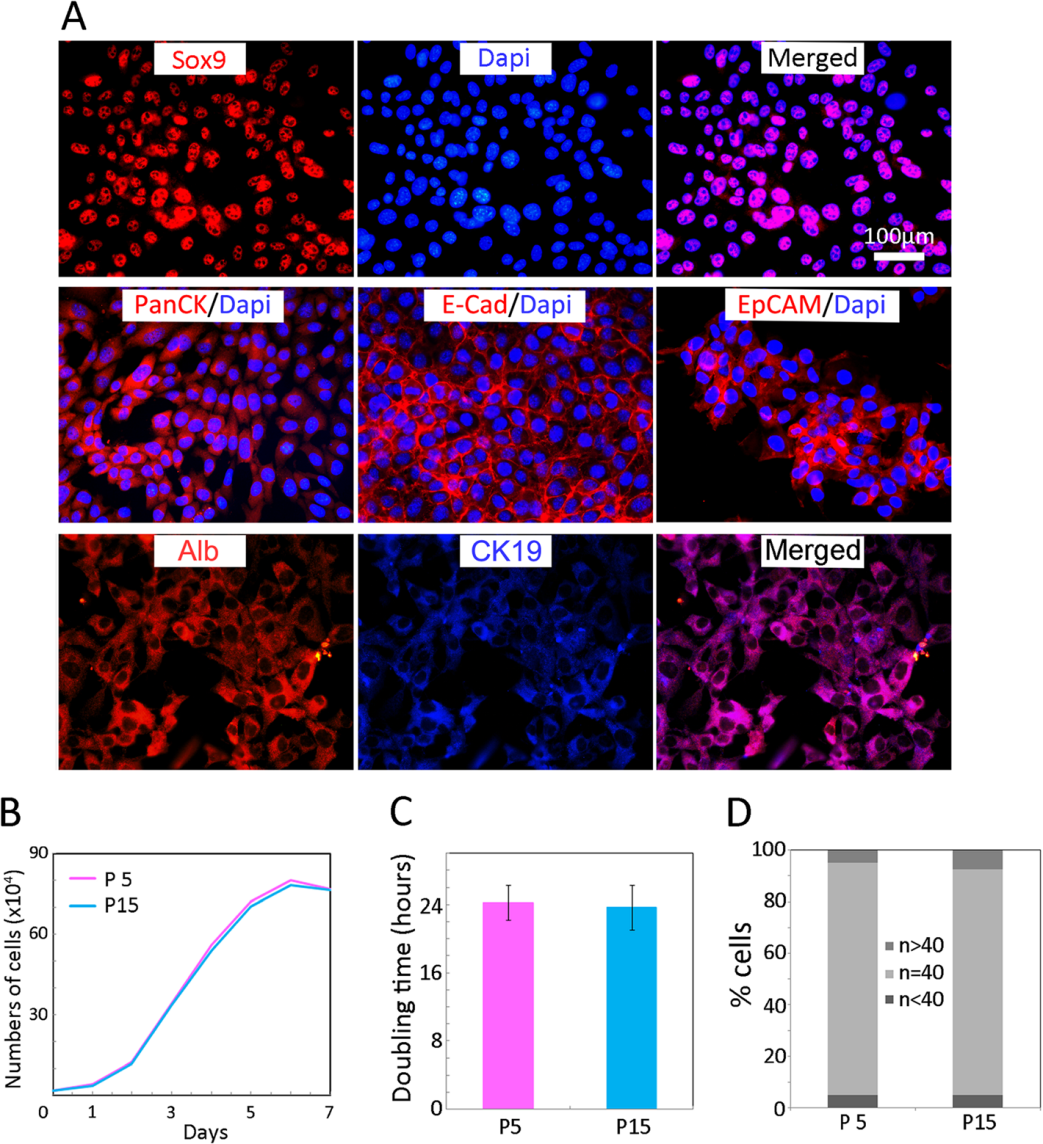
Characterization of CK19^+^ single-clone cells. **a** Immunostaining of representative single clone. Cells were stained with Sox9, Pan CK, E-Cad, EpCAM, Alb, and CK19 antibody. The nuclei were counterstained with DAPI. Scale bar, 100 μm. Growth curves (**b**) and doubling times (**c**) of passages 5 and 15 of a single clone. **d** The percentages of passage 5 cells with chromosomal counts *n* > 40, *n* = 40, and *n* < 40 chromosomal counts groups

Immunostaining confirmed that cells expressed hepatic stem cell-related markers: Sox9, Pan-CK, E-cadherin, EpCAM, albumin, and CK19 (Fig. 4a). These cells could be expanded for at least 15 passages and displayed a typical S-shaped growth curve (Fig. 4b), and the population doubling time was approximately 24 h (Fig. 4c). Karyotyping analysis revealed that most cells harbored normal chromosome numbers (Fig. 4d), indicating that the cells were genetically stable. Taken these together, CK19^+^ cells that were isolated from mouse gallbladder possessed hepatic stem cell characteristics.

### The combination of Noggin, inhibitors of ERK, and Hedgehog-induced gallbladder stem cells differentiated into insulin-secreting cells

Small molecules or recombinant proteins, which could regulate specific signal pathways, have been successively used for inducing embryonic stem cells or pluripotent stem cells toward pancreatic β-like cell fate [9–11] Based on previous studies, several key factors were chosen as candidates for inducing differentiation. Nicotinamide (Nico), an inhibitor of poly ADP-ribose synthetase, had shown an effect on the differentiation of fetal pancreatic tissue and stimulate proliferation of β cells [10]. Retinoic acid (RA) was proven to promote NGN^+^ cell differentiation to β cells [10, 11]. Treatment by an antagonist of bone morphogenetic proteins, Noggin, could promote definitive endoderm cells differentiating into pancreatic progenitor-like cells [9]. Cyclopamine (Cyclopa), one of the Hedgehog pathway inhibitors, was helpful to generate insulin-secreting cells from human embryonic cells [9]. ERK inhibitor (FR180204) and PI3K inhibitor (LY294002) both played an important role in regulating maturation of islet-like cells [12, 13]. Hepatocyte growth factor (HGF) could enhance insulin secretion [21, 22].

**Fig. 5:**
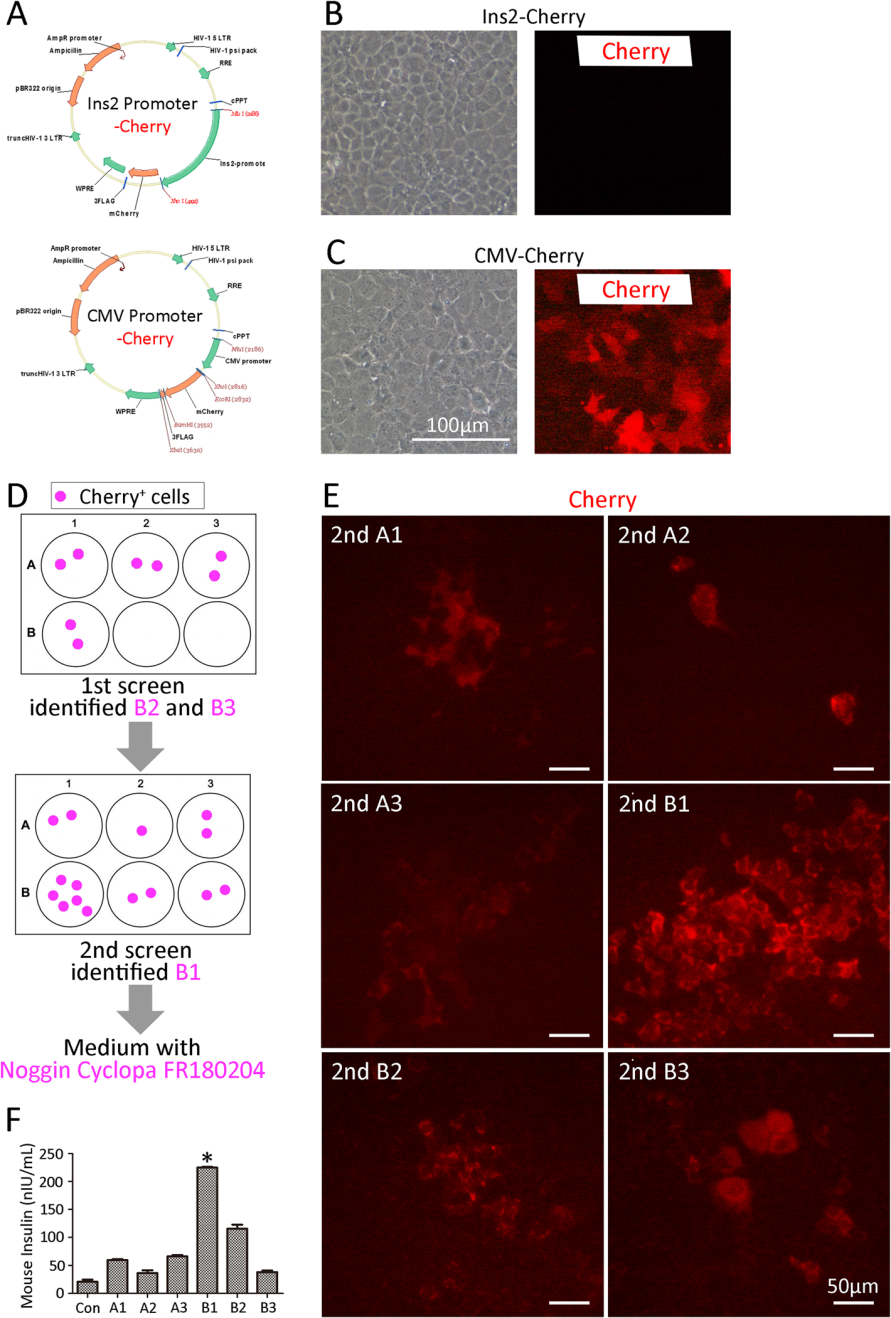
Induction of gallbladders stem cell differentiation into insulin-secreting cells in vitro. **a** Plasmid maps of the Insulin2 Promoter-Cherry and CMV Promoter-Cherry. **b**, **c** Phase contrast and red fluorescence view of gallbladder stem cells infected with the lentivirus. Scale bar, 100 μm. **d** Screening strategy to identify the best medium composition for induction. Pink round shape indicates Cherry^+^/insulin^+^ cells. **e** Representative images of cherry fluorescence in the second round screening. Scale bar, 50 μm. **f** Secreted insulin levels from each well of the second round screening. ^*^p < 0.05 (compared with each group)

To develop a strategy to differentiate gallbladder stem cells into insulin-secreting cells, we generated a Cherry-expressing cell line driven by the *insulin*-promoter (Fig. 5a, b) and devised a strategy to identify the most effective combination of the compounds (detailed in Additional file 1: Table S4). In the first round, the basic culture medium was supplemented with all the compounds, and different media with one reduced of each compound were added to the cells (Fig. 5d, top panel). In this round, we discovered that by reduction of Noggin and cyclopamine, no mCherry^+^ cells could be observed. This indicated that adding Noggin and inhibiting the Hedgehog pathway were the key elements. In the second round, the basic medium was supplemented with Noggin and cyclopamine. Then, the other five compounds were added to each well of cells (Fig. 5d, bottom panel). The results indicated that adding ERK inhibitor (FR180204) could significantly improve the number of insulin^+^ cells (Fig. 5e). Insulin-secreting levels in the culture supernatant of each group confirmed the results (Fig. 5f).

Taken together, our findings demonstrated that the combination of Noggin and inhibitors of the ERK and Hedgehog pathways could generate insulin-secreting cells from gallbladder stem cells.

### The insulin-secreting cells induced from the gallbladder exhibit characteristics of pancreatic beta cells

**Fig. 6:**
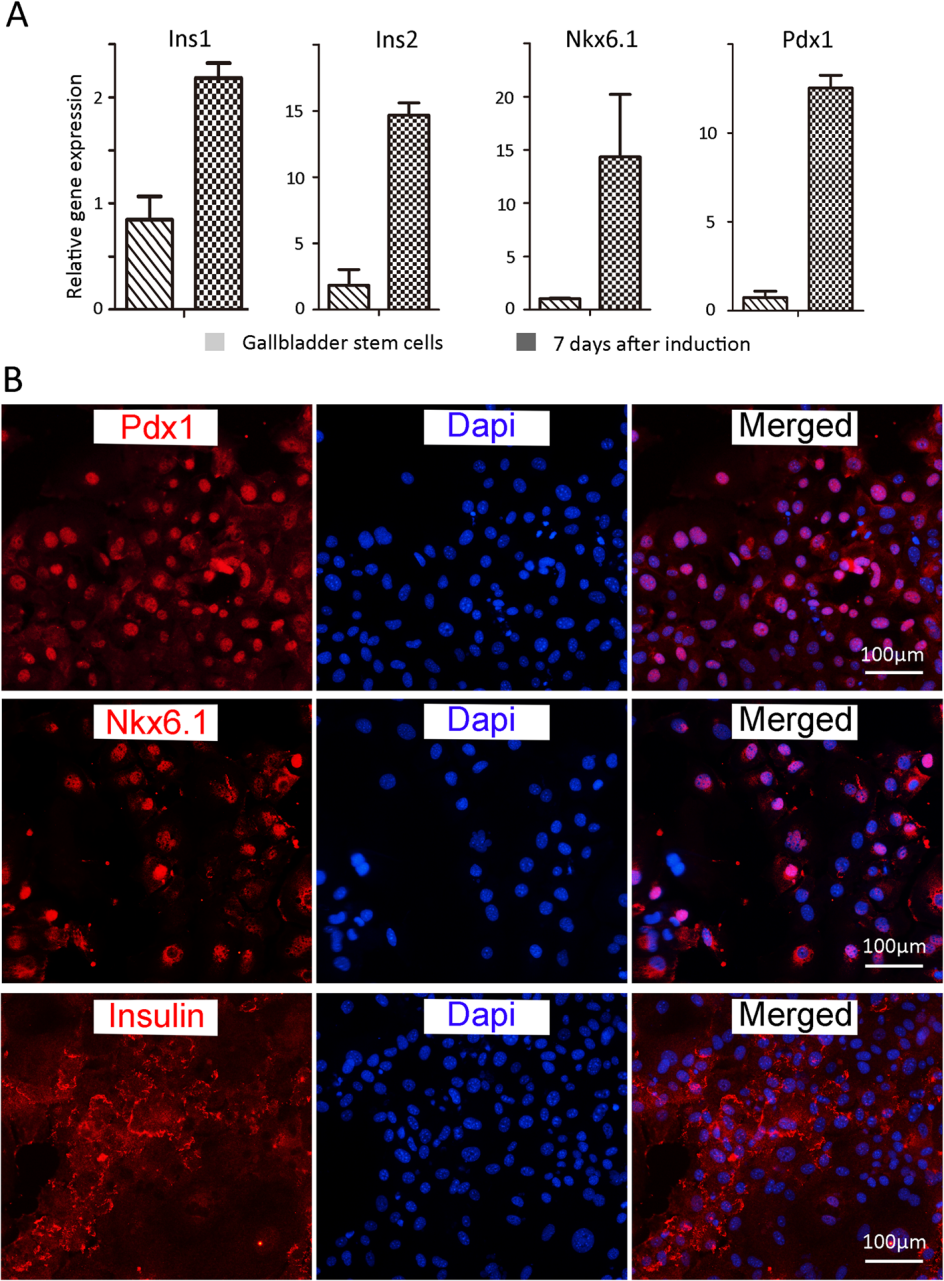
Characterization of gPB cells in vitro. **a** Gene expression analysis of pancreatic markers before and after the induction process by quantitative real-time PCR. The results were the average of three independent experiments. **b** Immunostaining of Pdx1, Nkx6.1, and insulin after differentiation. The nuclei were counterstained with DAPI. Scale bar, 100 μm

The gallbladder-derived insulin-secreting cells were first characterized with pancreatic genes, including key beta cell transcription factors: Pdx1 and Nkx6.1, as well as other endocrine markers: glucagon and somatostatin [5, 9, 11, 23]. The results showed that the cells were positive of key pancreatic cell markers: Pdx1 and Nkx6.1, and insulin 1 and 2, but were negative for GCG (Fig. 6a, Additional file 1: Figure S1). Then, these cells were stained with insulin, Pdx1, and Nkx6.1 antibody, confirming they exhibited characteristics of pancreatic beta-like cells (Fig. 6b). The efficiency of cells differentiated into insulin^+^ cells was 15.9% ± 1.2%. The insulin secretion level was 225 nIU/ml/day on average (the basic level of the medium was 20.98 ± 3.77 nIU/ml/day). Thus, our results demonstrated that this strategy could generate pancreatic beta-like cells, and we named these cells gallbladder-derived PB cells (gPB cells).

### gPB cells ameliorate hyperglycemia in diabetic mice after transplantation

**Fig. 7:**
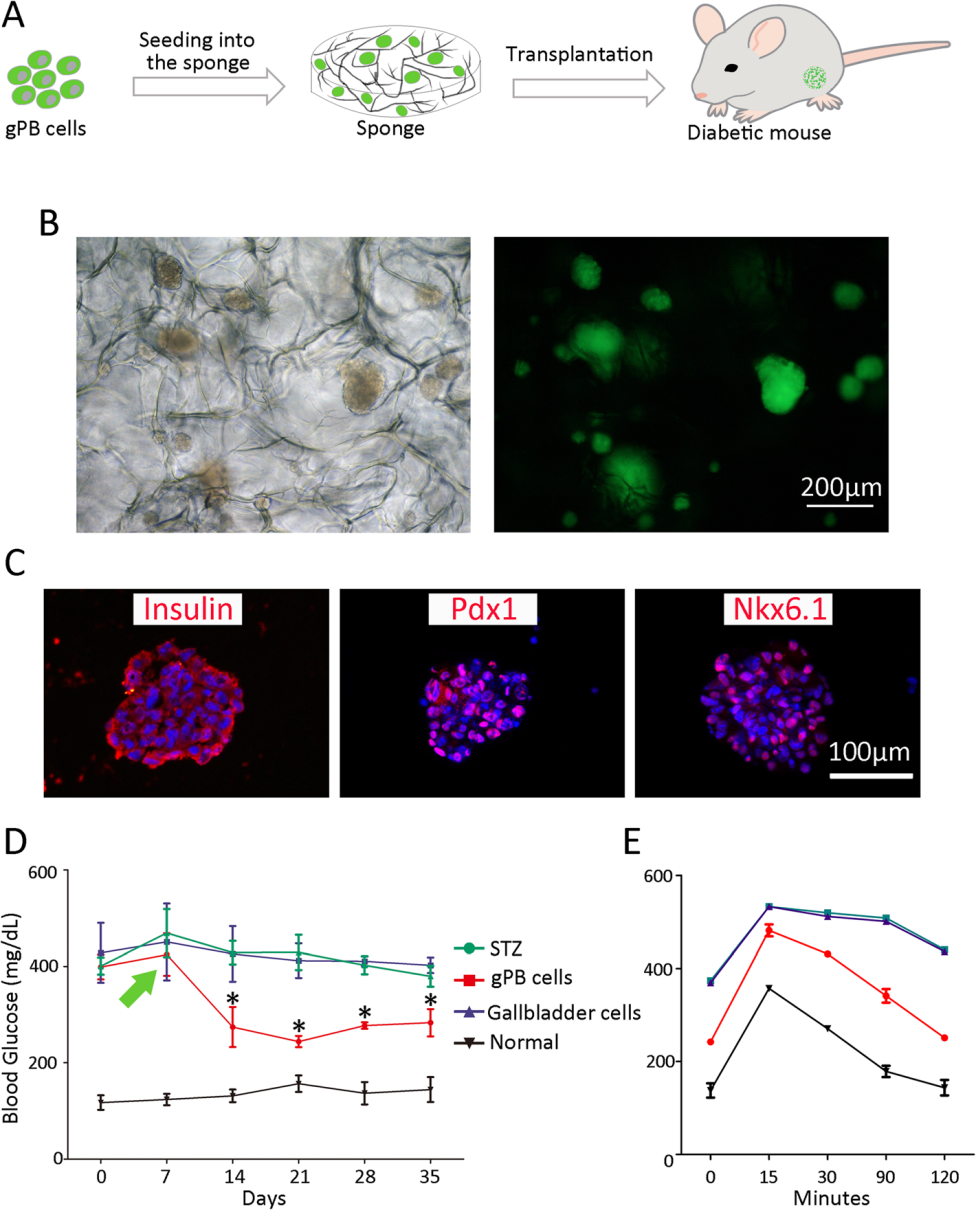
Characterization of gPB cells in vivo. **a** Schematic strategy of transplanted gPB cells. Cells were first seeded into the sponge and then put into epididymal pad site of diabetic mice. **b** Phase contrast and fluorescence images of the gPB cells after being seeded into the sponge. Scale bar, 200 μm. **c** Immunostaining of insulin, Pdx1, and Nkx6.1 after gPB cells are seeded into the sponge. The nuclei were counterstained with DAPI. Scale bar, 100 μm. **d** Monitoring of fasting blood glucose level of each group. STZ, diabetic mice without transplantation. gPB cells, diabetic mice were transplanted with gPB cells. Gallbladder cells, diabetic mice transplanted with gallbladder stem cells. Normal, normal mice. Green arrow indicates transplantation time. *p < 0.05 comparing the two cell groups (the gPB cells group and gallbladder cells group) on the same day with paired *t* test. Data presented as mean ± SEM. **e** Glucose tolerance tests of each group. Blood glucose was measured at 0, 15, 30, 90, and 120 min after glucose stimulation

To assess the function of gPB cells in vivo, we first seeded the gPB cells into the cellulosic sponge [24–26] and then put the sponge under the peritoneum [27] of STZ-induced diabetic mouse (Fig. 7a). The cellulosic sponge was a thick (1 mm thickness and 9 mm in diameter) and macroporous (200 μm average pore size) scaffold, which could maintain cell growing in spheroid with functionality [24–26]. After inducing gallbladder stem cell differentiation into gPB cells, they were digested and seeded into the sponge at a density of 5 × 10^5^. gPB cells immediately organized into 3D spheroids within 1 day of culture (Fig. 7b) and were positive for pancreatic markers (Fig. 7c, Additional file 1: Figure S2). Two days after seeding, the sponges were implanted at the epididymal pad site of diabetic mice, which were injected with STZ and became hyperglycemic before transplantation. In the normal (untreated) group, fasting blood glucose was unchanged during the observation period (135 ± 18 mg/dl). In the STZ (without cell transplantation) group, glucose levels increased and retained at a high level (> 400 mg/dl on average). In the group transplanted with uninduced gallbladder stem cells, glucose levels developed similarly to the STZ group. In the gPB cells transplanted group, glucose levels decreased 1 week after transplantation and maintained at the lower level (270 ± 27 mg/dl) compared to the STZ and gallbladder cell-treated group (Fig. 7d, green arrow indicated transplantation time). Furthermore, during the observation period, the gPB cell treatment could ameliorate polydipsia, polyphagia, and polyuria which are caused by diabetes. Four weeks post-transplantation, glucose tolerance test was conducted. The gPB cell treatment group could give rise to functional glucose-responsive (Fig. 7e) as measured by the levels of blood glucose.

These results indicated that gPB cells transplanted with the sponge could ameliorate hyperglycemia in a diabetic mouse model.

## Discussion

Our results demonstrate that insulin-secreting cells can be generated from gallbladder cells without genetic modification in vitro and they could be functional in vivo via transplantation. In addition, we provide a new approach for transplanting of endocrine hormone cells.

Diabetes, affecting more than 400 million people worldwide, is a kind of disease that may be treatable with cell transplantation [28]. Patients, especially with type 1 diabetes, can be made insulin independent for over 5 years through transplantation of new β cells. Thus, cell therapy holds great promise for millions of patients to treat diabetes [29, 30]. There were several kinds of cells that could potentially restore β cell function. Transplantation of mesenchymal stem cells was considered slowing the progression of diabetes and therefore allowing recovery of pancreatic cells. Also, β-like cells could be acquired from embryonic stem cell or induced pluripotent stem cells by stepwise differentiation [2, 3, 11, 29, 31–37]. Other studies demonstrated that cells from the liver, skin, etc. could be reprogrammed to β-like cells [30]. Potential problems of treating diabetes are safety, immunogenicity, and genetic instability of therapeutic cells. Further studies may concern these issues.

Some studies reported that liver progenitor cells or gallbladder epithelial cell could be reprogrammed to β cell fate [3, 7]. Ectopic or artificial expression of Pdx1, Ngn3, MafA, etc. by adenovirus was used to change cells’ fate. This genetically modified strategy was potentially risky and could not be applied in the clinical study. Small molecules have been widely utilized in manipulating stem cell fate or state [8, 9, 11]. Compared with genetic modification, regulation of cell fate by small molecules has significant advantages: it was specific, time-controllable, and reversible [8].

Our study has some obvious advantages. First, we provide a new approach of generating β-like cells from the gallbladder stem cells. The gallbladder is a highly available tissue, since it is discarded from donor livers, and a huge number of patients are subjected to cholecystectomy [3, 4, 7]. Moreover, diabetic patients could get insulin-secreting cells from their own gallbladder, which may alleviate immune rejection. Second, it takes 1 week to get the insulin-secreting cells by our approach, which is shorter than the others’ approaches (2 weeks on average). The reason may be that the gallbladder and ventral pancreas are both derived from the endoderm lineage. Thus, it is easier for gallbladder stem cells to change their fate. Additionally, we provide a new option for cell transplantation by the cellulosic sponge, which possesses well biological compatibility. β cells were usually embedded in the Matrigel and transplanted under the kidney capsule. This might cause unexpected injury to the body and cannot be applied to humans [9, 11, 23]. However, the sponge seeded with cells could be conveniently put into the body with minimal damage.

We notified that the glucose level of diabetic mice was significantly ameliorated after transplanted with gPB cells. However, Li et al. reported that the glucose level could further decrease to approaching the normal level by transplanting with reprogrammed pancreatic cells [9, 23]. It is possible that the number of transplanted cells that Li et al. used was 3 × 10^6^ per mouse, six times higher than what we used. Therefore, it is likely that increasing the number of transplanted cells using our method will generate better results. Also, Pagliuca et al. reported they could get functional β cells, which could flux Ca2^+^ and secrete insulin in response to glucose in vitro [11]. gPB cells we obtained in this study were treated with various differentiation factors for only 7 days, and they still proliferated well. Although gPB cells could secrete insulin, they cannot be called “functional β cells.” Further study is needed to explore strategies that could promote gPB cells maturation.

## Conclusion

In summary, we developed a convenient and rapid strategy to get insulin-secreting cells from gallbladder stem cells without gene modification. Also, we provide a new option for cell transplantation by the cellulosic sponge, with minimally invasive surgery. This provides an option for β cell therapy and drug testing in treating type 1 diabetes.

## Supplementary information

**Additional file 1:Table S1.** Primers list. **Table S2.** Primary antibodies. **Table S3.** Secondary antibodies. **Table S4.** The components of each well. **Figure S1.** gPB cells were negative for GCG. **Figure S2.** Gallbladder stem cells were negative for pancreatic markers. (DOCX 615 kb)

## Data Availability

The data that support the findings of this study are available from the corresponding authors upon reasonable request.

## Abbreviations

GSCs: Gallbladder stem cells
EpCAM: Epithelial cell adhesion molecule
GFP: Green fluorescent protein
CK19: Cytokeratin 19
Alb: Albumin
STZ: Streptozotocin
KM: Kubota’s medium
aSMA: Alpha-smooth muscle actin
Cyclopa: Cyclopamine
HGF: Hepatocyte growth factor
Gcg: Glucagon
Ins: Insulin
gPB cells: Gallbladder-derived pancreatic beta-like cells
