# Supplementary material for: Generation of insulin-secreting cells from mouse gallbladder stem cells by small molecules in vitro
Source: Stem Cell Res Ther. 2019 Sep 23;10:289. doi: 10.1186/s13287-019-1407-6 (PMC6757438; doi:10.1186/s13287-019-1407-6)
Supplement: Supplementary file 1 — Additional file 1:Table S1. Primers list. Table S2. Primary antibodies. Table S3. Secondary antibodies. Table S4. The components of each well. Figure S1. gPB cells were negative for GCG. Figure S2. Gallbladder stem cells were negative for pancreatic markers. (DOCX 615 kb) [file 13287_2019_1407_MOESM1_ESM.docx]

**Additional File Inventory**

Table S1, Primers list

Table S2, Primary antibodies

Table S3, Secondary antibodies

Table S4, The components of each well in the screening strategy

Figure S1, related to Figure 6

Figure S2, related to Figure 7

Additional file 1: Table S1 Primers list

| Gene | Forward(5’-3’) | Reverse(5’-3’) | Tm(℃) | Length |
| --- | --- | --- | --- | --- |
| Ins2 | ttgtcaagcagcacctttgt | ctgatctacaatgccacgct | 58 | 209 |
| Ins1 | cctgttggtgcacttcctac | agctccagttgttccacttg | 58 | 198 |
| Nkx6.1 | ttggcctattctctggggat | cattctccgaagtccccttg | 58 | 154 |
| Pdx1 | cacacagctctacaaggacc | gcacttcgtatggggagatg | 58 | 182 |
| Nkx2.2 | ggacaatgacaaggagaccc | gcacgtttcatcttgtagcg | 58 | 222 |
| Ngn3 | ttcgcccacaactacatctg | cttgggagactggggagtag | 58 | 157 |
| Gcg | agaaccccagatcattccca | tgttgttccggttcctcttg | 58 | 180 |
| Sst | aggacgagatgaggctgga | gaggtctggctaggacaacaa | 55 | 145 |
| Gapdh | aatggtgaaggtcggtgtga | cgctcctggaagatggtgat | 58 | 234 |

Table S2 Primary antibodies

| Antibody | Catalog | Source | Dilution | Company |
| --- | --- | --- | --- | --- |
| Insulin | MAB1417 | Rat polyclonal | 1:400 | R&D |
| Nkx6.1 | AF2517 | Goat polyclonal | 1:200 | R&D |
| PDX1 | AF5857 | Goat polyclonal | 1:200 | R&D |
| EpCAM | ab32392 | Rabbit polyclonal | 1:200 | Abcam |
| Sox9 | AB5535 | Rabbit polyclonal | 1:500 | Millipore |
| Krt19 | A3190 | Rabbit polyclonal | 1:400 | Abbomax |
| Albumin | Ab19194 | Rabbit polyclonal | 1:200 | Abcam |
| PanCK | sc-81714 | Mouse monoclonal | 1:200 | Santa Cruz |
| E-cadherin | ab40772 | Rabbit polyclonal | 1:200 | Abcam |
| aSMA | ab28052 | Mouse monoclonal | 1:200 | Abcam |

Table S3 Secondary antibodies

| Antibody | Catalog | Dilution | Company |
| --- | --- | --- | --- |
| Alexa Fluor® 594 Donkey Anti-Rat IgG | 1398009 | 1:400 | Molecular  Probes |
| Alexa Fluor® 488 Donkey Anti-Rat IgG | 1744717 | 1:400 | Molecular  Probes |
| Alexa Fluor® 594 Donkey Anti-Goat IgG | 1608643 | 1:500 | Molecular  Probes |
| Alexa Fluor® 488 Donkey Anti-Goat IgG | AB150129 | 1:400 | Abcam |
| Alexa Fluor® 568 Donkey Anti-Rabbit IgG | A10042 | 1:800 | Molecular  Probes |
| Alexa Fluor® 488 Donkey Anti-Rabbit IgG | A21206 | 1:800 | Molecular  Probes |
| Alexa Fluor® 568 Donkey Anti-Mouse IgG | A10037 | 1:800 | Molecular  Probes |

Table S4 The components of each well

| **The first round** | | | | | | | |
| --- | --- | --- | --- | --- | --- | --- | --- |
|  | Nicotinamide | LY294002 | Cyclopamine | Noggin | FR180204 | Retinoic acid | |
| A1 | + | - | + | + | + | + | |
| A2 | + | + | + | + | + | + | |
| A3 | + | + | + | + | + | - | |
| B1 | + | + | + | + | - | + | |
| B2 | + | + | + | - | + | + | |
| B3 | + | + | - | + | + | + | |
| **The second round** | | | | | | | |
|  | Nicotinamide | LY294002 | Cyclopamine | Noggin | FR180204 | HGF | RA |
| A1 | - | + | + | + | - | - | - |
| A2 | + | - | + | + | - | - | - |
| A3 | - | - | + | + | - | + | - |
| B1 | - | - | + | + | + | - | - |
| B2 | - | - | + | + | - | - | + |
| B3 | - | - | + | + | - | - | - |
| A1-B3 refers to culture media with different combination of candidate molecules. | | | | | | | |

Figure S1


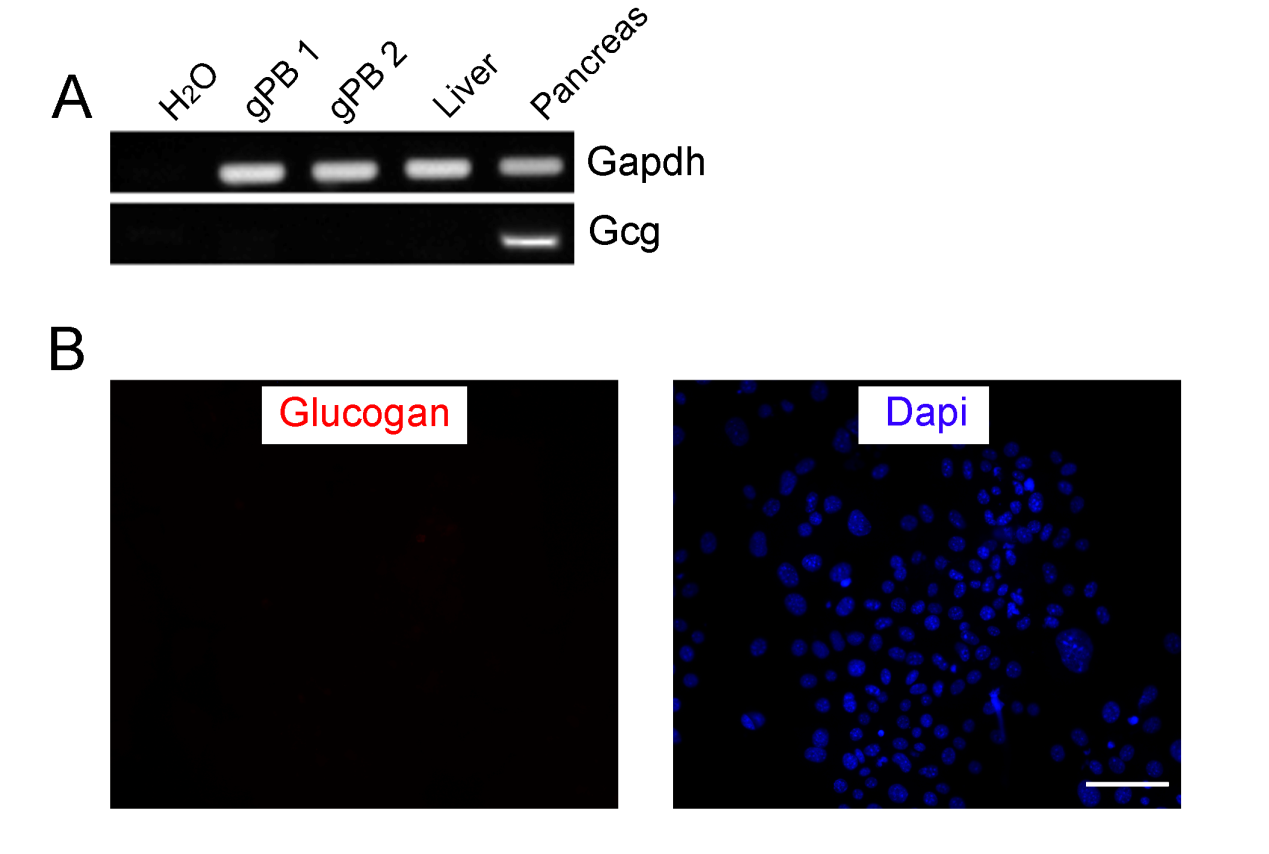


**Figure S1** gPB cells were negative for GCG

A RNA expression results for detection of Glucogan (GCG). B Immunostaining of GCG in the gPB cells. Nuclei were counterstained DAPI. Scale bar, 100μm.

6. Supplemental Figure 2


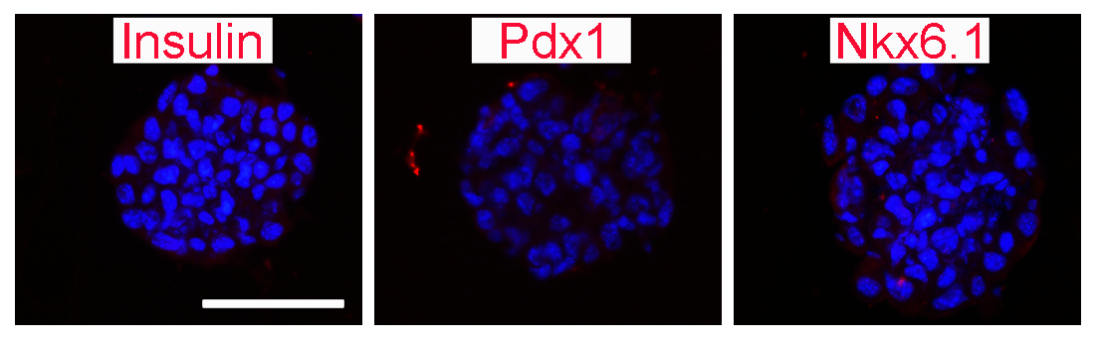


**Figure S2** Gallbladder stem cells were negative for pancreatic markers

Immunostaining of Insulin, Pdx1 and Nkx6.1 after gallbladder stem cells seeded into the sponge. Nuclei were counterstained DAPI. Scale bar, 100μm.
